# Topical Pirfenidone-Loaded Liposomes Ophthalmic Formulation Reduces Haze Development after Corneal Alkali Burn in Mice

**DOI:** 10.3390/pharmaceutics14020316

**Published:** 2022-01-28

**Authors:** Carlos Daniel Diaz-Palomera, Isaac Alejandro Vidal-Paredes, Jose Navarro-Partida, Margarita Cid-Hernandez, Luis Carlos Rosales-Rivera, Ricardo De la Rosa-Bibiano, Hugo Christian Monroy-Ramirez, Arturo Santos, Juan Armendariz-Borunda

**Affiliations:** 1 Instituto de Biologia Molecular en Medicina y Terapia Genica, Centro Universitario de Ciencias de la Salud, Universidad de Guadalajara, Guadalajara 44340, Jalisco, Mexico; daniel.qfb.farma@gmail.com (C.D.D.-P.); ricardo_rb12@hotmail.com (R.D.l.R.-B.); christian.monroy0981@cucs.udg.mx (H.C.M.-R.); 2 Tecnologico de Monterrey, Escuela de Medicina y Ciencias de la Salud, Monterrey 64849, Nuevo Leon, Mexico; isaac.vidal@tec.mx (I.A.V.-P.); josenavarro@tec.mx (J.N.-P.); 3 Centro de Retina Medica y Quirurgica, S.C., Centro Medico Puerta de Hierro, Zapopan 45116, Jalisco, Mexico; 4 Departamento de Quimica, Centro Universitario de Ciencias Exactas e Ingenierias, Guadalajara 44430, Jalisco, Mexico; margarita.cid@academicos.udg.mx; 5 Departamento de Ingenieria Quimica, Universidad de Guadalajara, Guadalajara 44430, Jalisco, Mexico; carlos.rosales@academicos.udg.mx

**Keywords:** cornea, haze, pirfenidone, liposomes, nanoparticles

## Abstract

Corneal chemical burns (CCBs) frequently result in corneal fibrosis or haze, an opacity of the cornea that obstructs vision and induces corneal blindness. Diverse strategies have been employed to prevent or reduce CCB-related corneal haze. In this study, we evaluated the physicochemical characteristics and biologic effects of a topical pirfenidone (PFD)-loaded liposomal formulation (PL) on a corneal alkali burn mice model. We found that PL was appropriate for ocular application due to its physiologic tear pH, osmolarity and viscosity suitable for topical ophthalmic use. Regarding its therapeutic activity, PL-treated mice had significantly reduced haze size and density, corneal edema, corneal thickness, and corneal inflammatory infiltration, in contrast to PFD in aqueous solution (*p* < 0.01). Importantly, the antifibrotic activity of PL (reduction of corneal haze) was associated with modulation of transforming growth factor (TGF)-β and Interleukin (IL)-1β genes. PL suppressed TGF-β expression and restored normal IL-1β expression in corneal tissue more efficiently in contrast to PFD in aqueous solution. In conclusion, PFD showed essential anti-inflammatory and anti-fibrotic effects in the treatment of alkali burns. Noteworthy, a new formulation of PFD-loaded liposomes remarkably improved these effects, standing out as a promising treatment for corneal haze.

## 1. Introduction

The cornea is the most important refractive structure in the eye. Loss of corneal transparency is the second leading cause of blindness worldwide, only after cataracts [1]. Chemical corneal burns (CCBs) are some of the most common workplace-related accidents and account for about 11–22% of all ocular injuries, though, children among 1–2 years old are a high-risk group [2,3]. CCBs frequently result in corneal fibrosis or haze, a cloudy, opaque appearance of the cornea, that obstructs the vision and induces corneal blindness [4].

Corneal alkali burns are more common and severe than acid burns. Generally, acid substances tend to coagulate proteins in the corneal epithelium, thus limiting their penetration into the eye, whereas alkali substances penetrate easier and deeper, spreading the damage. Corneal healing following alkali burns rarely restores the transparency and culminates in corneal haze and opacity [1,2,3,5,6]. Corneal myofibroblasts, α-smooth muscle actin (αSMA)-positive cells, are the main cells involved in corneal haze development [7] and are generated from corneal keratocytes through a transforming growth factor β (TGF-β)-mediated epithelial-mesenchymal transition process, triggering αSMA expression and increasing collagen I secretion [8,9].

Because of the challenge of treating corneal blindness secondary to CCBs, multiple efforts have been made to design effective treatment options capable to modulate corneal wound healing and to prevent or reduce corneal haze. Among these, topical ophthalmic formulations containing innovative therapeutic agents stand out: (1) a formulation containing the dominant negative survivin protein (SurR9-C84A) and histone deacetylase inhibitor trichostatin-A (TSA) [10], (2) a formulation with the verbenone derivative SP-8356, an inhibitor of the cluster of differentiation 147 (CD147), also known as extracellular matrix-metalloproteinase (MMP) inducer (EMMPRIN) [11], (3) an ophthalmic formulation based on nanostructured lipid carriers containing rapamycin [12] and (4) a pirfenidone ophthalmic formulation [13]. Hence, it is a clear rationale that topical formulations being in contact with the injured ocular surface for a lengthy period of time, and that can increase tissue penetration, will have a better chance of success in the CCBs treatment.

Pirfenidone (PFD) is an antifibrotic, antioxidant and anti-inflammatory agent with therapeutic activity in different organs and tissues, such as lung, kidney, and liver, downregulates TGF-β and collagens expression, and lessens cell proliferation and migration [14,15,16,17]. Regarding the use of PFD for CCBs, there are still unsolved concerns about the drug’s short half-life after its topical application in rabbit corneas. Due to dynamic barriers in the precorneal area such as tear turnover and lacrimal drainage via the nasolacrimal drainage system, the water-based PFD eye drops exhibited a short half-life and poor bioavailability [18]. Consequently, several strategies have been developed to increase PFD bioavailability in the cornea and conjunctiva to achieve a needed therapeutic effect in CCBs. One of them is the use of viscosity-enhancers such as hydroxypropyl methylcellulose to extend the ocular residence time of PFD. This approach has been recognized to prolong the residence time from 10 min to more than 20 min resulting in increased PFD levels in cornea, conjunctiva, sclera and aqueous humor until 90 min after topical administration [19]. Additionally, the use of nanotechnology is a promising strategy that has proven to increase the bioavailability and the therapeutic effect of PFD. For example, PFD-loaded poly lactic-co-glycolic acid (PLGA) nanoparticles significantly reduced collagen I level, corneal haze and the time for corneal re-epithelialization following alkali burn in rats, as opposite to free pirfenidone [20]. Thus, it is obvious to reason that a PFD formulation increasing corneal bioavailability will have the best chance to succeed.

Liposomes, small vesicles (approximate size of 30–1000 nm) which are prepared with phospholipids, offer an efficient option for PFD drug delivery. Previously, liposomes have been evidenced to increase drug penetration, extend the time in contact with the ocular surface, and to improve the therapeutic effect of topical ophthalmic drugs, thus reducing the frequency of application [21,22]. We anticipate that due to these nanoparticles, PFD penetration will increase the time of interaction with the ocular surface and would improve the therapeutic effect by reducing the frequency of application. In this study, we evaluated the biological effect of PFD-loaded liposomes in a mice model of corneal alkali burn.

## 2. Materials and Methods

### 2.1. Preparation of PFD-Loaded Liposomes Formulation

Pirfenidone-loaded liposomes (PL) were generated based on our previous experience with ocular liposomal formulations. In a previous report, five different formulations were tested to deliver and release triamcinolone acetonide into the eye [23]. From these formulations, the best performance in terms of pH, osmolarity, viscosity, stability and encapsulation efficiency was related to a composition similar to the one described here in Table 1. Therefore, we decided to test the ability of this formulation to generate pirfenidone-loaded liposomes. Self-forming and thermodynamically stable PFD-loaded liposomal formulation (PL) was formed as previously described [23,24]. Briefly, PFD (Tecoland Corporation, Irvine, CA, USA) was first added to a lipid mixture containing polyethyleneglycol (PEG-12, Sigma-Aldrich, St. Louis, MO, USA) glyceryl dimyristate and ethyl alcohol (Sigma-Aldrich, St. Louis, MO, USA). An aqueous mixture containing grade 2 purified water, polyethylene glycol (15)-hydroxystearate (Kolliphor HS 15, Sigma-Aldrich, St. Louis, MO, USA), citric acid anhydrous (Sigma-Aldrich, St. Louis, MO, USA), sodium citrate dehydrate (Sigma-Aldrich, St. Louis, MO, USA), and benzalkonium chloride (Sigma-Aldrich, St. Louis, MO, USA) was commingled in a flask and set aside for compounding at room temperature. The water mixture was gently added to the lipid mixture to obtain the final formulation. PL was filtered through 0.22 mm pore size membranes (Merck Millipore, Billerica, MA, USA) under aseptic conditions. The final composition of PL is described in Table 1. Final PFD concentrations in the ensuing dispersion were 0.2 mg/mL (0.02%) and 1 mg/mL (0.1%).

### 2.2. Characterization of PFD-Loaded Liposomes Formulation

Physicochemical and microscopic characterization of PFD formulation was carried out. Firstly, morphology of PFD in aqueous solution, in ethanolic solution and in liposomes was explored through scanning electron microscopy (SEM) and transmission electron microscopy (TEM). Aqueous and ethanolic solutions of PFD were prepared by adding ultrapure water or ethanol to the required quantity of PFD crystals to achieve the concentrations of 0.1%. For SEM analysis, a TESCAN MIRA3 LMU FE-SEM device was used (Tescan Orsay Holding, a.s., Brno-Kohoutovice, Czech Republic), while for TEM studies a JEOL JEM-1010 electron microscope was used (Jeol USA, Peabody, MA, USA). SEM samples were kept at −4 °C before being mounted onto stubs and gold-coated using a Denton Vacuum Desk II sputter coater (SPI supplies, West Chester, PA, USA). TEM samples were previously treated using phosphotungstic acid as a negative staining agent in a 1:1 dilution (*v*/*v*) and were deposited onto FF 300 square mesh copper grids (Electron Microscopy Sciences, Fort Washington, PA, USA) for observation. Manual counting and measurement of particles were performed using SEM micrographs at a view field of 63.6 mm to calculate the size and distribution of PLs.

Finally, the physicochemical properties, size distribution, and zeta potential of different PL formulations and diluted samples were determined at 33 °C, which is the ocular surface temperature [25]. The osmolality of 10 µL of sample was measured at room temperature using a Vapro 5600 vapor pressure osmometer (ELITechGroup, Paris, France). The viscosity of PL formulations was measured at a shear rate of 100 s^−1^ using a stress-controlled AR-G2 rheometer (TA Instruments, New Castle, DE, USA) with a 60 mm cone-and-plate geometry of 2° angle. pH was monitored using an Orion Star A210 (Thermo Fisher Scientific, Waltham, MA, USA). Intensity-sized distributions, polydispersity index (PdI), and zeta potential values of diluted liposomal formulations in double-distilled water (ddH_2_O) or 1 mM PBS buffer (pH 7.4) were determined via dynamic light scattering (DLS) in a Zetasizer Nano ZS90 (Malvern Instruments, Malvern, UK). DLS measurements were performed using a dispersant refractive index of 1.33 and an absorption index of 0.01. Zeta potential values were obtained using the same diluted samples in a disposable capillary cell (DTS1070). All experiments were performed in triplicate.

### 2.3. Evaluation of the Antifibrotic Activity of PFD in Human Corneal Fibroblasts (HCFs)

Evaluation of the antifibrotic activity of PFD was carried out in primary cell culture of human corneal fibroblasts (HCFs). HCFs were obtained from donor corneal transplant remnant tissue according to Guo X, et al. [26]. Concisely, corneal remnants were carefully scraped on both sides with a scalpel, removing epithelium and endothelium, cut into small pieces and cultured in a 6-well cell culture plate using Dulbecco’s Modified Eagle’s medium (DMEM, Gibco, Life Sciences, Grand Island, NY, USA) containing 10% fetal bovine serum (FBS, Gibco, Life Sciences, Grand Island, NY, USA) and 1% antibiotic-antimycotic (Gibco, Life Sciences, Grand Island, NY, USA) at 37 °C in 5% CO_2_. Once cells reached confluency, HCFs were detached using TrypLE Express Reagent (Gibco, Life Sciences, Grand Island, NY, USA) and subcultured in T75 cell culture flask for posterior use. Experiments were carried out with HCFs on passage 4. To evaluate the antifibrotic effect of pirfenidone in vitro, we seeded 1 × 10^6^ cells into a T75 cell culture flask. Once cells were at 60–70% confluency, DMEM was changed to serum-free culture media for 72 h before adding TGF-β1 (5 ng/mL) with or without PFD (100 µM) for 24 h (TGF-β1 and TGF-β1 + PFD 100 µM groups, respectively). The expression of pro- and anti-fibrogenic genes was explored in cell cultures by qRT-PCR and cell immunofluorescence assays.

The methodology for RT-PCR assays is described below. Total RNA was extracted from HCFs using TRIzol reagent (Invitrogen, Carlsbad, CA, USA) according to the manufacturer’s indications. Cells were lysed adding TRIzol and mixing by pipetting up and down, followed by the addition of chloroform. The aqueous phase containing RNA was separated and mixed with isopropanol and kept at −20 °C, overnight. The mixture was centrifuged at 12,000 RCF for 20 min at 4 °C. The RNA pellet was washed using cold ethanol 70% *v*/*v* and resuspended in 40 µL of RNase free water. cDNA was obtained using the High-Capacity cDNA Reverse Transcription Kit (Applied Biosystems, Foster City, CA, USA) according to the manufacturer’s indications. RT-PCR was performed using TaqMan Universal PCR Master Mix and TaqMan probes for αSMA, Col1a1, Col3a1 and Col5a1 (Applied Biosystems, Foster City, CA, USA), 18s gene was used as endogenous control. Relative quantification by 2^−ΔΔCT^ method was performed using the TGF-β1 group as internal calibrator [27,28].

Additionally, cell immunofluorescence assays were carried out in HCFs exposed to PFD. Briefly, HCFs were seeded (1 × 10^5^ cells) on coverslips placed in 6-well cell culture plates, followed by the addition of DMEM containing 10% FBS and 1% antibiotic-antimycotic. Once cells reached confluency, media was changed to serum-free media for 72 h. Subsequently, media was changed to DMEM containing TGF-β (5ng/mL) with and without PFD (100µM) (TGF-β1 and TGF-β1 + PFD 100 µM groups, respectively) for 24 h at 37 °C in 5% CO_2_. After incubation, cells were fixed using 4% paraformaldehyde for 15 min, followed by 3 washes with PBS. Cells were blocked and permeabilized using blocking buffer (5% albumin, 0.1% Triton X-100) for 1 h. Cells were incubated with anti-αSMA primary antibodies (Cell Signaling, Danvers, MA, USA) at 4 °C overnight. Cells were washed with PBS three times and incubated with secondary antibody (Alexa Fluor Goat anti-rabbit IgG, Cell Signaling) for 2 h. Nuclei were stained with 4′,6-diamidino-2-phenylindole (DAPI, Invitrogen, Carlsbad, CA, USA). Samples were examined under a confocal microscope (Leica Microsystems, Wetzla, Germany).

### 2.4. Evaluation of the Therapeutic Activity of PFD in a Mice Mode of Corneal Alkali Burn

To induce the corneal alkali burn mice model, the right cornea of thirty-five male C57BL/6 mice was exposed to NaOH (Sigma-Aldrich, St. Louis, MO, USA). First, animals were anesthetized using intraperitoneal Ketamine/Xylazine (40 mg/kg/5 mg/kg, PiSA Farmaceutica, Guadalajara, Mexico) and 2 tetracaine drops to the experimental eye. Subsequently, filter paper of approximately 2 mm of diameter soaked with 0.5 M NaOH was placed on the cornea (right eye) for 30 s and the eye was then washed immediately with abundant sterile saline solution. Then, animals were assigned to 7 groups (5 mice per group): (1) PBS treated eyes (PBS), (2) Empty liposome treated eyes (EL), (3) Dexamethasone treated eyes (DEX), (4) PFD treated eyes with PFD in aqueous solution at 0.02% (0.02% PFD), (5) PFD treated eyes with PFD in aqueous solution at 0.1% (0.1% PFD), (6) PFD treated eyes with PFD in liposomes at 0.02% (0.02% PL) and (7) PFD treated eyes with PFD in liposomes at 0.1% (0.1% PL). Additionally, 5 mice with non-burned eyes (NB) were used as healthy controls.

Treatments with PFD in aqueous solution, PFD contained in liposomes (0.02% and 0.1%), as well as empty liposomes and dexamethasone (1%), were instilled 4 times per day for 15 days in the right cornea. At the end of the treatment, all mice were sacrificed by an overdose of pentobarbital (100 mg/kg, PiSA Farmaceutica, Guadalajara, Mexico). Whole eyes were fixed in 4% paraformaldehyde (Sigma-Aldrich, St. Louis, MO, USA) overnight, and later, parallel sections were made, paraffin blocks were prepared and multiple slides were obtained from each block. Slides were stained by routine hematoxylin and eosin (H&E) staining. These slides were used to evaluate inflammation, edema and corneal thickness.

Single-blind analysis of edema, inflammation and corneal thickness was performed using Image Pro Plus 6.0 software (Media Cybernetics Inc., Rockville, MD, USA). Edema was measured by quantifying blank pixels between collagen fibers of the corneal stroma, which correspond to the areas with edema. Subsequently, all colored pixels that make up the corneal tissue were quantified. The percentage of empty pixels within the cornea corresponds to tissue edema. Inflammation was assessed by manually identifying and measuring areas with inflammatory infiltrate of mononuclear and polymorphonuclear cells, which are distinguishable from the spindle morphology of corneal fibroblasts. The percentage of area with inflammatory infiltrate within the entire cornea corresponds to tissue inflammation. Corneal thickness was measured at the central cornea using ImageJ software (http://imagej.nih.gov/ij/index.html accessed on 11 March 2021; provided in the public domain by the National Institutes of Health, Bethesda, MD, USA).

Additional corneal tissue slides were used for immunofluorescence analysis to identify fibrosis marks. Briefly, corneal tissue slides were deparaffinized in xylene, rehydrated in a series of graded alcohol, boiled in 10 mM sodium citrate buffer pH 6.0 for 10 min, cooled and washed with distilled water. Slides were blocked and permeabilized using blocking buffer (5% albumin, 0.1% Triton X-100, Sigma-Aldrich, St. Louis, MO, USA) for 1 h, and incubated with anti-αSMA, anti-TGF-β and anti-Interleukin-1β (anti-IL1β) primary antibodies (Cell Signaling) at 4 °C overnight. After 3 washes with PBS, the slides were incubated with a fluorescent secondary antibody (Alexa Fluor Goat anti-rabbit IgG or anti-mouse IgG, Cell Signaling) for 2 h. Nuclei were stained with DAPI. Samples were examined under a confocal microscope (Leica Microsystems).

### 2.5. Statistical Analysis

Data are presented as mean ± standard deviation. Data analysis was performed using GraphPad Prism statistical software (GraphPad Software Inc., San Diego, CA, USA) by one-way analysis of variance (ANOVA) followed by a Dunnet post hoc test or Kruskal–Wallis test when appropriate. Statistical significance was defined as a *p* value less than 0.05.

### 2.6. Ethical Considerations

This study was approved by the research ethics committee of Centro Universitario de Ciencias de la Salud, Universidad de Guadalajara (reference number: Cl-00321, approved on 28 January 2021). Animal housing, care, and application of experimental procedures were all carried out in accordance with the ARVO Statement for the Use of Animals in Ophthalmic and Vision Research, as well as the Mexican Official Standard NOM-062-ZOO-1999. Animals were sacrificed following the AVMA Guidelines for the Euthanasia of Animals (2013 edition).

## 3. Results

### 3.1. PFD Is Efficiently Loaded in Liposomes and It Is Suitable for Topical Ophthalmic Use

**Figure 1:**
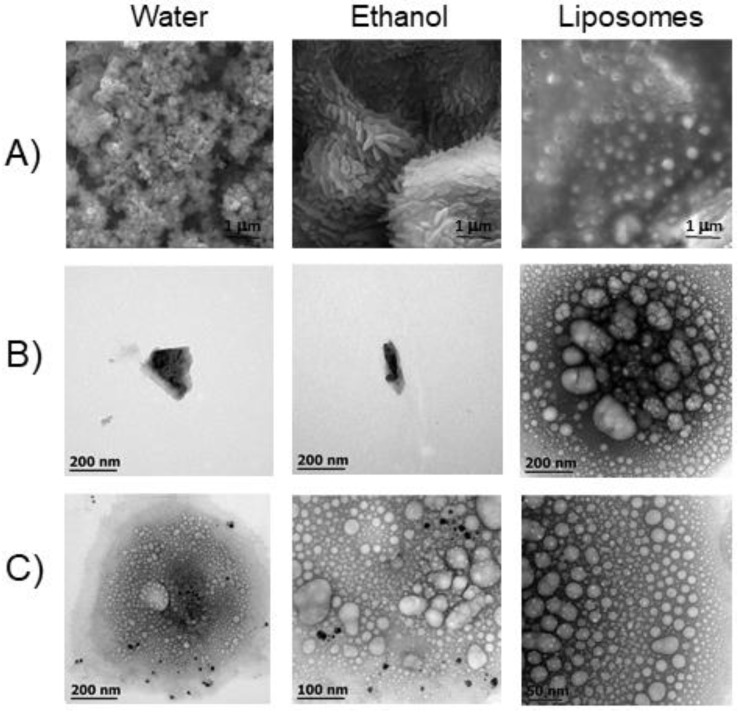
Liposomal encapsulation of pirfenidone. SEM and TEM images of PFD crystals in liposomes formulation, ethanolic and aqueous solutions. (**A**) SEM shows that PFD crystal shape is modified by ethanolic solution, whereas liposomal formulation completely dissolves it. (**B**) TEM images reveal that PFD crystal size is reduced by ethanol solution, compared with aqueous solution while PFD crystals are observed inside liposomes. (**C**) TEM images of liposomes containing PFD crystals are presented in different magnifications. PFD, pirfenidone; SEM, scanning electron microscopy; TEM, transmission electron microscopy.

SEM and TEM examination of PFD-loaded liposome (PL) formulations revealed interesting findings (Figure 1). PFD crystal structure is altered when in solution with ethanol, whereas it is completely dissolved by the liposomal formulation, as observed by SEM. TEM images revealed that PFD crystal size was smaller in ethanol solution than in aqueous solution, and PFD crystals were seen inside liposomes.

On the other hand, the average particle size and PdI of diluted PL formulations was 263 ± 10 nm for 0.1% liposomes (1/200 *v*/*v*) and 0.37 ± 0.04 in 1 mM PBS buffer, respectively. In ddH_2_O, the average particle size (256 ± 2.6 nm) and PdI (0.28 ± 0.01) recorded were slightly lower, both with a uniform sample distribution. For 0.02% liposomes, the average particle size was 214 ± 2.8 nm, with a PdI value of 0.29 ± 0.03 in PBS, while in ddH_2_O the particle size was 253 ± 5.0 nm, with a PdI value of 0.35 ± 0.01. The average zeta potential measured in PBS buffer was −20.4 ± 0.1 and −20.9 ± 0.7 mV for 0.1% and 0.02% liposomal formulations, respectively. Both PL formulations had a negative surface charge. It is important to emphasize that, although negative and neutral charged liposomes in ocular systems are easily drained from the precorneal area due to the negative charge of the corneal epithelium [29], the viscosity of PL samples at 33 °C (discussed in the next section) was similar to a soft gel. Therefore, it is expected that this characteristic increases the residence time of the formulation at the ocular surface, and its bioavailability in consequence [30].

In ddH_2_O, the average zeta potential for liposomes 0.1% and 0.02% was −26.6 ± 0.7 and −19.4 ± 0.4, respectively.

Overall, the pH of PL formulations was acidic, with values of 5.72 and 6.06 for 0.1% and 0.02% PL formulations, respectively, at 33 °C. The physiologic human tear pH range is located between 6.5 to 7.6, but pH values in the range of 4–8 are well tolerated by the eye. Regarding viscosity, the results obtained herein proved that a maximum increase of penetration through the cornea by an eye drop solution takes place when viscosity falls into the range of 15 to 150 mPa·s [31]. The viscosity at a shear rate of 100 s^−1^ for 0.02% PL and 0.1% PL at 33 °C was 42.2 and 32.9 mPa·s, respectively. Osmolarity values were similar for both PL formulations (~100 mmol/kg). A substance is considered non-irritating to the eye when the osmolarity values fall within 205 and 684 mmol/L. However, hypotonic formulations can be found in dry eye formulations and no adverse reactions to hypotonic solutions have been observed. Complete size distribution, zeta potential, and physicochemical characterization of PL are shown in Table 2.

### 3.2. PFD Reduces Expression of Pro-Fibrogenic Genes in HCF Primary Cell Culture

**Figure 2:**
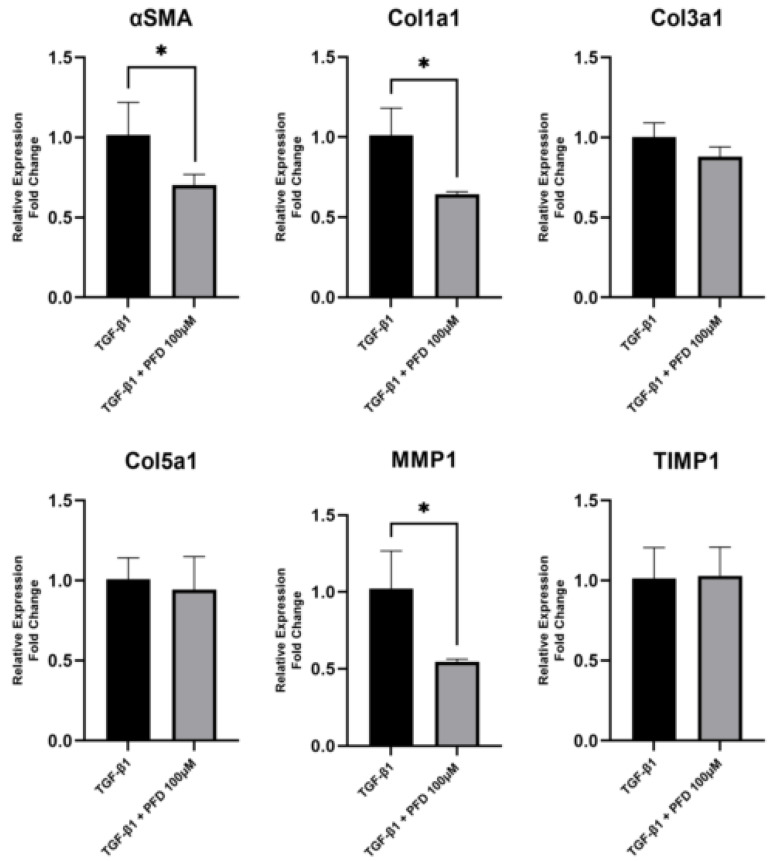
Gene expression of fibrosis-related genes in primary HCF cell culture in response to treatment with TGF-β and pirfenidone. HCF cell were stimulated with TGF-β for 24 h in the presence or absence of PFD at 100 µM. Gene expression was measured by RT-PCR. PFD at 100 µM reduced αSMA, MMP1 and Col1a1 mRNA expression. Data represented as mean ± standard deviation. HCF, human corneal fibroblast; TGF-β, transforming growth factor-β; PFD, pirfenidone; TIMP1, tissue inhibitor matrix metalloproteinase 1; αSMA, α smooth muscle actin; Col1A1, collagen 1A1; Cola3A1, collagen 3A1; Col5A1, collagen 5A1; MMP1, matrix metalloproteinase 1; RT-PCR, real time polymerase chain reaction. * *p* < 0.05 vs. TGF-β1 group.

**Figure 3:**
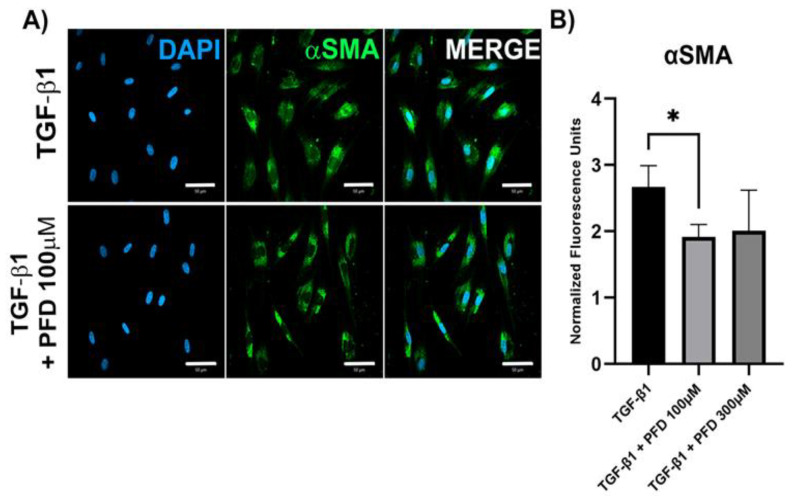
Analysis of αSMA protein expression by immunofluorescence in primary HCF cell culture in response to treatment with TGF-β and pirfenidone. HCF cells were stimulated with TGF-β for 24 h in presence or absence of PFD at 100 µM. (**A**) Immunofluorescence images obtained by confocal microscopy. Scale bar 10 µm for all panels. (**B**) Semi-quantification by fluorescence intensity analysis. Data expressed as mean ± standard deviation. αSMA, α smooth muscle actin; DAPI, 4′,6-diamidino-2-phenylindole; TGF-β, transforming growth factor-β; HCF, human corneal fibroblast; PFD, pirfenidone. * *p* < 0.05.

To determine the effect of different PFD concentrations in HCF primary cell culture, we evaluated mRNA expression of fibrosis-related genes after 24 h of TGF-β stimulation with or without PFD at 100 µM by RT-PCR (Figure 2). We found that PFD at 100 µM significantly reduces the expression of αSMA (*p* < 0.05), a main myofibroblast marker, as well as Col1a1 (*p* < 0.05) and MMP1 (*p* < 0.05). Meanwhile, expression of TIMP1, Col3a1 and Col5a1 was barely or non-affected by PFD. When evaluating αSMA protein expression by immunofluorescence, we found that 100 µM PFD significantly reduced αSMA protein quantity (*p* < 0.05) (Figure 3).

### 3.3. PFD Reduces Corneal Haze, Inflammation and Edema after Alkali Burn

**Figure 4:**
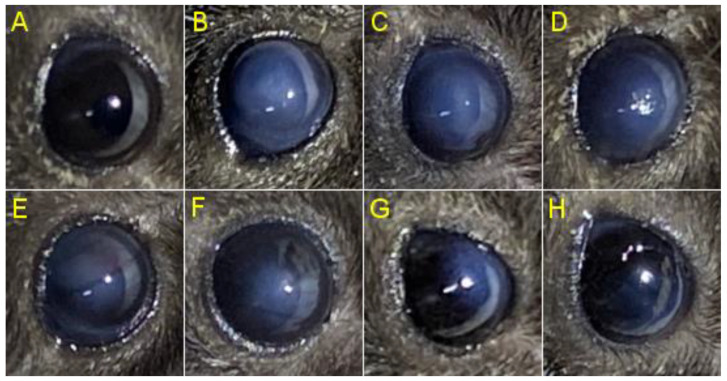
Comparison of corneal haze induced by alkali burn after 15 days of treatment. Representative photographs from the different experimental groups are presented and are as follow; (**A**) non-burned eye, (**B**) PBS treated eye, (**C**) empty liposome treated eye, (**D**) dexamethasone treated eye, (**E**) 0.02% PFD treated eye, (**F**) 0.1% PFD treated eye, (**G**) 0.02% PL treated eye and (**H**) 0.1% PL treated eye. PFD (**E**,**F**) and PL (**G**,**H**) treated eyes have a greater reduction in haze extension and density than those eyes treated with PBS (**B**), empty liposomes (**C**) and dexamethasone (**D**). PBS, phosphate buffered saline; PFD, pirfenidone; PL, pirfenidone-loaded liposome.

**Figure 5:**
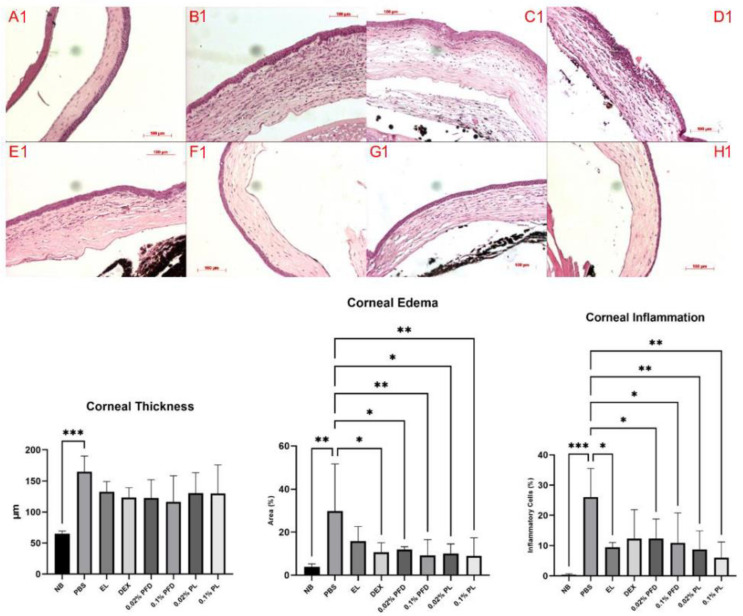
Evaluation of corneal thickness, edema and inflammation in corneal tissue. (**A**) Representative microphotographs from the different experimental groups are presented and are as follows A1. non-burned eye (NB), B1. PBS treated eye (PBS), C1. empty liposome treated eye (EL), D1. dexamethasone treated eye (DEX), E1. 0.02% PFD treated eye, F1. 0.1% PFD treated eye, G1. 0.02% PFD-loaded liposomes (PLs) treated eye and H1. 0.1% PL treated eye. (**B**) Quantitative analysis of corneal thickness, edema and corneal inflammation. When comparing PBS group with NB group, a significant increase of corneal thickness, edema degree and inflammatory infiltrated cells quantity is evident. PFD decreased edema and reduced corneal thickness. Additionally, PL had a more significant reduction of corneal inflammation (*p* < 0.01) than PFD at matched concentrations (*p* < 0.05). Data expressed as mean ± standard deviation. NB, non-burned; PBS, phosphate-buffered saline; EL, empty liposome; DEX, dexamethasone; PFD, pirfenidone; PL, pirfenidone-loaded liposome. * *p* < 0.05 vs. PBS group; ** *p* < 0.01 vs. PBS group; *** *p* < 0.001 vs. PBS group.

After 15 days of treatment of the mice model of corneal chemical injury, we observed a reduction in haze size and density in the treated eyes which was more evident in those treated with PFD (Figure 4). Histology analysis (Figure 5) showed an increase in corneal thickness of about 100 µm in the PBS group compared with non-burned (NB) corneas (*p* < 0.01), meanwhile treatment groups reduced this thickening by about 50 µm compared with the PBS group. Additionally, we evaluated the degree of edema and infiltration of inflammatory cells in the corneal stroma. We observed that the PBS group had an evident increase in corneal edema compared with the NB group (*p* < 0.01). However, treatments including dexamethasone, PFD and PL showed a significant decrease in corneal edema escalation. Finally, with the purpose of evaluating inflammation, we determined the inflammatory cells infiltrated in the corneal tissue. An evident increase of inflammatory cells in corneal tissue of the PBS group was observed when compared with the NB group (*p* < 0.001), nevertheless, PFD (*p* < 0.05) and PL (*p* < 0.01) showed a significant reduction of inflammatory infiltrated cells in corneal stroma. These results show PDF anti-inflammatory effect and its biologic action enhancement by liposomes.

### 3.4. PFD and PFD-Loaded Liposomes Suppress αSMA Expression in Corneal Tissue

**Figure 6:**
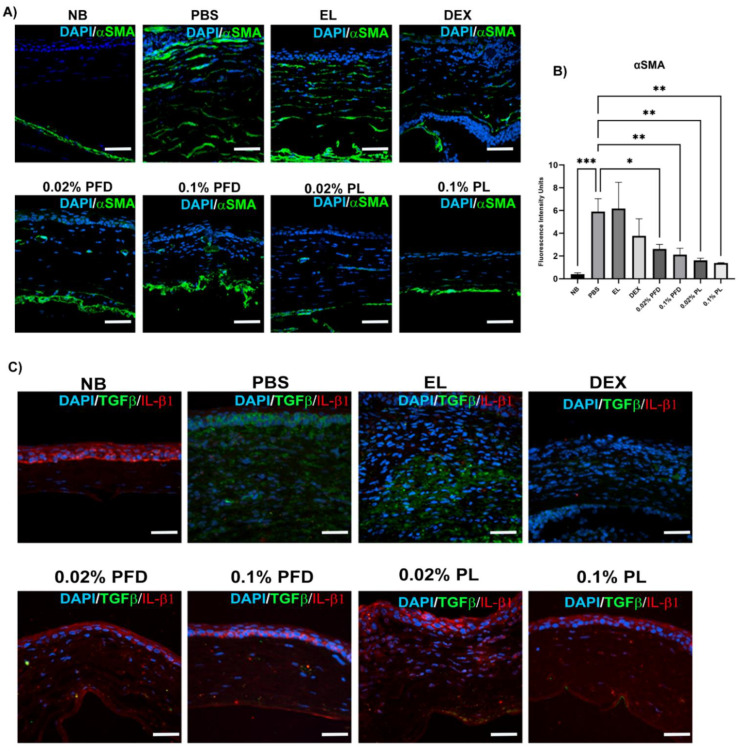
Comparison of αSMA, TGF-β and IL-1β expression in corneal tissue among different treatments by immunofluorescence. (**A**) PFD suppresses αSMA in a dose-dependent manner, and liposomes improve its effect. Representative immunofluorescence images obtained by confocal microscopy. (**B**) Semi-quantification of αSMA protein expression by fluorescence intensity analysis. Data expressed as mean ± standard deviation. (**C**) PFD suppresses TGF-β in a dose-dependent manner and liposomes improve this effect. Meanwhile, PFD restores normal epithelial location of IL-1β. Non-burned eyes (NB), PBS treated eyes (PBS), empty liposome treated eyes (EL), dexamethasone treated eyes (DEX), 0.02% PFD treated eyes, 0.1% PFD treated eyes, 0.02% PFD-loaded liposome (PL) treated eyes and 0.1% PL treated eyes. αSMA, α smooth muscle actin; TGF-β, transforming growth factor-β; IL-1β, interleukin-1β; DAPI, 4′,6-diamidino-2-phenylindole; NB, non-burned; PBS, phosphate-buffered saline; EL, empty liposome; DEX, dexamethasone; PFD, pirfenidone; PL, pirfenidone-loaded liposome. * *p* < 0.05 vs. PBS group; ** *p* < 0.01 vs. PBS group; *** *p* < 0.001 vs. PBS group. Comparison of corneal haze induced by alkali burn after 15 days of treatment.

Increased expression of αSMA is a clear sign of a fibrogenic process, consequently, αSMA is one of the main profibrotic biomarkers. Immunofluorescence image analysis shows that NB corneas do not express αSMA. Meanwhile, the PBS group shows an evident rise in αSMA expression. However, PFD showed a significant suppression in αSMA expression in a dose-dependent manner. Additionally, PL improved the effect of PFD, inducing a further decrease of αSMA expression (Figure 6A,B).

### 3.5. PFD and PFD-Loaded Liposomes Suppress TGF-β Expression in Corneal Tissue and Restores Normal IL-1β Expression

TGF-β is one of the most important pro-fibrogenic cytokines; an increased expression of this cytokine induces myofibroblast transformation and collagen I secretion. Besides, IL1-β, a pro-inflammatory cytokine secreted after corneal damage, regulates myofibroblast presence by inducing apoptosis of resident fibroblasts. In immunofluorescence images, we observed an increment of TGF-β expression in PBS, EL and dexamethasone (DEX) groups compared with NB group. Nevertheless, PFD suppressed TGF-β expression in a dose-dependent manner, while PL notoriously enhanced this effect at matched doses. On the other hand, IL-1β appeared to be confined to the interior of corneal epithelial cells of NB corneas. PBS, EL and DEX groups show loss of epithelial localization and expression of IL-1β, as well as presence in the corneal stroma. Noteworthy, PFD and PL restored expression and epithelial localization of IL-1β (Figure 6C).

## 4. Discussion

Ocular chemical burn outcome depends largely on the clinical management provided. Current treatments consist of immediate and continuous irrigation, promotion of re-epithelization, suppression of inflammation and prevention of complications. Artificial drops, topical corticosteroids and ascorbate are the main treatment options for chemical burn management; however, prolonged use of topical corticosteroids may lead to the development of sterile ulcers [3,32]. Nevertheless, this treatment scheme does not contemplate the stromal fibrotic response.

Corneal transparency depends mainly on stromal structure. Corneal stroma is composed of about 90% of extracellular matrix (ECM), principally collagen. This collagen is regularly packed into small diameter (~25 nm) fibrils packed as lamellae, this arrangement minimizes light scattering and permits transparency. Corneal collagen fibrils are composed mostly of collagen I and lesser amounts of collagen V, as well as some proteoglycans. These features allow collagen fibrils to maintain their diameter and separation [33].

Corneal injury triggers a complex series of processes, known as wound healing response, whose purpose is to restore the normal structure and function of the cornea. However, an abnormal wound healing response leads to loss of corneal transparency or haze development due to myofibroblast transformation, the crucial factor that leads to corneal stromal fibrosis [4]. Results shown here demonstrate that our therapeutic strategy might impede myofibroblast transformation and, thus, prevent corneal haze formation after CCBs.

Myofibroblasts, characterized by αSMA expression, are highly contractile cells able to synthesize and deposit great amounts of ECM components, especially collagen I and III, changing collagen diameter fibrils and disarranging stromal structure [34,35]. The cornea does not have myofibroblasts present under normal conditions, they derive from resident keratocytes after injury in response to TGF-β1 released by epithelial cells [36].

PFD has shown anti-fibrotic effects reducing TGF-β1 expression and ECM deposit in different organs [17]. Different preclinical studies have exhibited PFD therapeutic potential in the treatment of fibrotic and proliferative eye disorders including corneal chemical burns [13], conjunctival scarring [37,38,39], and choroidal neovascularization (CNV) [40]. It has been documented that PFD reduces αSMA expression, promotes epithelial restoration and suppresses infiltration by inflammatory cells in rat corneas [13]. Our results showed a reduction in the expression of collagen I and III, αSMA, MMP1 and TGF-β1 in the model of chemical corneal injury, making evident the PFD anti-fibrotic effect. In addition, PLs decreased inflammation by reducing inflammatory cells influx to the corneal stroma.

On the other hand, myofibroblast presence seems to be regulated by IL-1β secretion. This cytokine released by epithelial cells triggers apoptosis of myofibroblasts when TGF-β1 is withdrawn from the cells [4,34,36]. IL-1β is constitutively expressed by corneal epithelial cells and is released in minimal amounts but does not pass into the stroma in the absence of injury [41,42]. Interestingly, in non-burned corneas we can observe IL-1β located exclusively in epithelium, but in PBS, EL and DEX groups, this expression is lost and decreases its corneal presence. Noteworthy, PL treatment groups restored IL-1β expression to nearly normal conditions, reestablishing the pro-apoptotic effect that this cytokine has on myofibroblasts, as well as displaying an appropriate reinstatement of the corneal epithelium.

In spite of that, PFD shows a short half-life in the cornea after topical application, anti-fibrotic and anti-inflammatory effects in corneal alkali burns are notorious [37]. Moreover, the use of liposomes remarkably improved these effects. The use of new delivery systems such as liposomes is part of novel strategies to avoid early degradation of drugs and regulate their release into the stroma, reducing the frequency of application [21,22,43].

Different polymeric, non-polymeric and lipid-based nanosystems have been proposed to be administered topically to deliver drugs into the eye. For example, PFD in the nanoparticles were tested previously in an alkali burn mice model. This system was able to reduce the TGF-β and αSMA expression, as well as collagen I secretion [20]. In contrast, liposomes constitute a lipid-based nanosystem that has shown a high efficiency over the last years, making them a target for constant and thorough research. Liposomes can transport hydrophilic, hydrophobic, and amphipathic drugs, with a greater improvement when using a lipophilic drug-like PFD. Liposome formulations have the advantage to be biodegradable with a relatively non-toxic behavior, which enhances drug permeation by binding to the corneal surface [22]. Therefore, use of liposomes to deliver PFD increases the therapeutic activity of the drug as we demonstrated here.

## 5. Conclusions

Current clinical management of corneal alkali burns is focused on promoting re-epithelization and reducing inflammation, but not on preventing haze development. PFD demonstrated its notorious anti-inflammatory and anti-fibrotic effects in the treatment of alkali burns. Moreover, PFD-loaded liposomes remarkably improved these effects, standing out as a promising treatment for the treatment of corneal fibrosis.

Thus, in a still-changing world, it is obvious to reason that drug formulations lasting longer in contact with damaged surfaces will have the best chance to succeed.

## Figures and Tables

**Table 1:** Pirfenidone-loaded liposomes formulation composition.

Reagent	Volume
Pirfenidone	1 or 0.2 mg
Kolliphor HS 15	50 mg
Polyethylene Glycol (PEG)-12 glyceryl dimyristate	100 mg
Ethyl alcohol	14 µL
Citric acid anhydrous	0.8 mg
Sodium citrate dihydrate	4.675 mg
Benzalkonium chloride	0.1 mg
Grade 2 purified water	Q.S.1.0 mL

**Table 2:** Physicochemical properties, size distribution and zeta potential of PL formulations.

Formulation	pH	Viscosity (mPa·s)	Osmolarity (mmol/kg)	Diluted PL (1/200 *v*/*v*)	Size (d.nm)	PdI	ζ (mV)
PL 0.1%	5.72	32.9	103.38 ± 10.15	PL 0.1% (PBS)	263 ± 10	0.37 ± 0.04	−20.4 ± 0.2
PL 0.02%	6.06	42.2	101.47 ± 9.16	PL 0.02% (PBS)	214 ± 2.8	0.29 ± 0.03	−20.9 ± 0.7
				PL 0.1 (ddH_2_O)	256 ± 2.6	0.28 ± 0.01	−26.6 ± 0.7
				PL 0.02% (ddH_2_O)	253 ± 5.0	0.35 ± 0.01	−19.4 ± 0.9

The values represent the average of three measures. PdI = polydispersity index, ζ = zeta potential.

## Data Availability

The data presented in this study are available on request from the corresponding author.
